# Long-Term Potentiation and Neurotransmitter Expression Change in Dysautonomia Linked to Binge Eating Disorder: Protective Role of Exercise

**DOI:** 10.3390/biology14101410

**Published:** 2025-10-14

**Authors:** Fernanda Veladiz-Gracia, Diana Elinos, Constanza González-Sierra, Angel Rubio-Galicia, Fredy Cifuentes, Miguel Angel Morales

**Affiliations:** Departamento de Biología Celular & Fisiología, Instituto de Investigaciones Biomédicas, Universidad Nacional Autónoma de México, Ciudad de México 04510, Mexico; veladizfernanda@gmail.com (F.V.-G.); diana.elinos@iibiomedicas.unam.mx (D.E.); cmarygonsierra@gmail.com (C.G.-S.); arubiogalicia@gmail.com (A.R.-G.)

**Keywords:** synaptic plasticity, superior cervical ganglia, autonomic, sympathetic, eating behavior, co-transmission/segregation

## Abstract

**Simple Summary:**

The autonomic nervous system is the branch of the nervous system that controls internal organs, keeping them in stable balance known as homeostasis. It can adapt its structure and function to meet new demands, a property known as plasticity. When it fails, a condition called dysautonomia occurs, which can arise on its own or be linked to diseases such as binge eating disorder. This disorder involves the repeated, compulsive eating of large amounts of high-calorie food in a short time. In our study, we recreated this disorder in rats to understand how it affects autonomic function and whether exercise has a positive effect. We studied possible changes in neuroplasticity in sympathetic ganglia, particularly in long-term potentiation of nerve transmission and in expression of neurotransmitters. Binge eating was induced through cycles of food restriction and free feeding, combined with access to tasty high-caloric food and frustration stress. We found neuroplasticity changes, including reduced potentiation, increase in one neurotransmitter, and altered neurotransmitter distribution. Exercise prevented the development of binge eating and avoided these nerve changes. Data could help to diagnose binge eating disorder as a form of dysautonomia, improve understanding of its symptoms and body-wide effects, and support exercise as a protective, non-drug intervention.

**Abstract:**

The autonomic nervous system (ANS) regulates internal organ function to maintain homeostasis. Dysautonomias are ANS disorders involving reduced or excessive sympathetic or parasympathetic activity and can be associated with metabolic syndrome and eating disorders such as binge eating disorder (BED). The ANS exhibits synaptic plasticity phenomena, including long-term potentiation (LTP) and neurotransmitter expression changes, which may influence autonomic function. BED is defined as recurrent, compulsive intake of large amounts of high-calorie food in a short time. Here, we examined dysautonomia in a rat BED model induced by cycles of food restriction and access to highly caloric food, and assessed whether exercise prevents these alterations. After confirming BED induction, we characterized LTP in the superior cervical ganglion (SCG) and analyzed acetylcholine (ACh) and GABA expression and their co-localization/segregation. BED rats exhibited impaired LTP and increased GABA expression. Voluntary aerobic exercise prevented BED onset and the associated changes in LTP and GABA. We propose that BED-associated dysautonomia proceeds at least in the ganglionic sympathetic cholinergic transmission, with reduced sympathetic activity. These results may contribute to a better understanding of the autonomic disorder associated with BED and support exercise as a protective intervention.

## 1. Introduction

The autonomic nervous system (ANS), composed of sympathetic, parasympathetic, and enteric divisions, regulates a wide range of involuntary physiological processes, including heart rate, blood pressure, and metabolism, among others [1]. These functions are modulated throughout the day depending on the physiological condition of the organism, such as periods of rest, stress, or physical activity.

The ANS exhibits forms of synaptic plasticity that contribute to the control and regulation of target organs. Synaptic plasticity may be involved in the modulation of regular functions by enhancing tonic efferent impulses directed toward peripheral targets. One form of synaptic plasticity described in sympathetic ganglia is long-term potentiation (LTP), particularly in the superior cervical ganglion (SCG) [2,3]. Another aspect of ANS regulation involves neurotransmitter expression, especially the balance between colocalization and segregation of transmitters. We have shown that neurotransmitters can be segregated into distinct synaptic boutons within the SCG [4]. Furthermore, we have demonstrated that the segregation of sympathetic neurotransmitters, acetylcholine (ACh), met-enkephalin, and GABA, undergoes plastic changes in response to variations in neurotrophin levels [5], stress [6], and aging [7].

Dysautonomia, or autonomic dysfunction, refers to alterations in the function of the autonomic nervous system. These alterations may involve either increased or decreased activity of the sympathetic or parasympathetic divisions, as well as imbalances between them. The most common indicators of dysautonomia include elevated plasma or urinary norepinephrine levels, increased muscle sympathetic nerve activity (MSNA), elevated blood pressure, and altered heart rate variability [1,8,9]. Dysautonomia has been identified in several pathological conditions, including hypertension, stress, [3,6], metabolic syndrome (MS) [10,11], and eating behavior disorders (EBDs) [12,13].

One of the most prevalent EBDs worldwide is binge-eating disorder (BED), which is defined as the recurrent, compulsive consumption of large quantities of food, primarily high-calorie, in a short period of time [14]. Despite its high prevalence, BED remains underdiagnosed and undertreated [15]. Individuals affected by BED have been reported to exhibit dysautonomia, characterized by reduced parasympathetic activity [12] and increased sympathetic function [13]. Compared to obese individuals without BED, patients with BED show greater vulnerability to stress, partly due to impaired cardiovascular adaptation to stressors. This is reflected in elevated heart rate (HR) reactivity and reduced heart rate variability (HRV) [14]. As with other human disorders, various experimental animal models have been developed to facilitate the study of BED [16]. These models often involve protocols such as alternating cycles of food restriction and access to high-caloric diets, sometimes in combination with acute or repeated stress [17].

Exercise contributes to the restoration of homeostatic control and to promote a rebalancing of sympathetic and parasympathetic activity [18,19]. Protective effects of exercise have also been reported in experimental models of high-fat diet intake and binge-eating disorder [20,21].

Among the various strategies used to evaluate autonomic function and characterize dysautonomia, we have focused on neurotransmission and its plasticity, as well as on the expression of neurotransmitters in specific efferent pathways. In SCG, we have investigated LTP and neurotransmitter expression under different physiological and pathological conditions. Previous studies from our group demonstrated increased basal sympathetic transmission and enhanced LTP in hypertension [3], along with alterations in neurotransmitter presence and changes in the balance between colocalization and segregation in conditions such as stress, hypertension, and aging [6,7].

Particularly in BED, it is widely accepted that the relationship between sympathetic activity and the disorder remains unclear, and that further research is necessary to better understand this interaction [22,23]. To contribute to clarifying this matter, in the present study we investigated whether dysautonomia associated with BED changes sympathetic activity and plasticity by assessing possible alterations in LTP and neurotransmitter expression in the SCG. Additionally, we examined the potential modulatory effect of exercise on these alterations.

Considering that BED has become a serious medical problem, preclinical models are necessary to investigate the neurobiology and psychobiology of binge eating, as well as to evaluate pharmacological strategies to identify effective treatments. Rats are the most used experimental animals. Accordingly, we used a rat model of BED, similar to that described by Romano et al., 2020 [24], which involved repeated cycles alternating unrestricted and restricted food access, including periods of access to high-caloric food (HCF), followed by exposure to stress. To assess the effect of physical activity, half of the consumed-HCF animals were given free access to a running wheel throughout the model induction period.

## 2. Methods

### 2.1. Animals

Eighteen standard male Wistar rats, 8 weeks old and weighing 250–300 g, were used. All procedures were conducted in accordance with the ethical guidelines for the care and use of laboratory animals established by the National Academy of Sciences of the United States. The study was approved by the Committee for the Care and Use of Laboratory Animals (CICUAL) of our Institute. Every effort was made to minimize both the number of animals used and any stress associated with handling.

### 2.2. Induction of the Experimental Model

**Figure 1:**
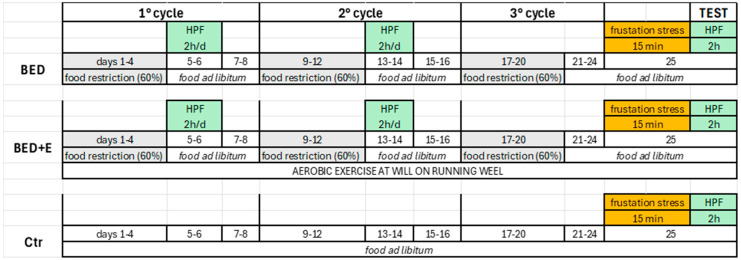
Protocol for inducing the binge eating disorder (BED) model in rats. Three groups were used: BED, BED plus exercise (BED+E), and control (Ctr). Eight-week-old male rats in the BED and BED+E groups underwent three 8-day cycles of intermittent food restriction (60% of baseline chow intake) on days 1–4, followed by free feeding (chow) on days 5–8. On days 5–6 and 13–14 of the first two cycles, rats were given access to highly caloric food (HCF) for 2 h during the light phase (13:00–15:00 h). In the third cycle (days 21–24), no HCF was provided. On the test day (day 25), all groups were maintained on free feeding. BED+E rats had ad libitum access to voluntary aerobic exercise throughout the protocol. Ctr rats received chow ad libitum for the entire duration. At 13:00 h on the test day, all groups underwent a 15-min frustration stress, consisting of visual and olfactory exposure to inaccessible HCF. Immediately after, rats had free access to HCF for 2 h, followed by chow ad libitum until tissue collection for electrophysiological and immunohistochemical analyses.

To induce the binge-eating disorder (BED) model, a protocol based on restricted food access, similar to that described by Romano et al., 2020 [24], was implemented. Rats received either standard chow pellets (Tekland, Envigo; 3.1 kcal/g) or a highly palatable high-caloric food (HCF; Oreo cookies: 4.8 kcal/g; 5.2% protein, 21.3% fat, and 73.5% carbohydrates), along with free access to water. Animals were randomly assigned to one of three groups (*n* = 6 per group): control (Ctr), BED, and BED plus exercise (BED+E). Rats were housed in pairs in plastic cages (46 × 24 × 20 cm) and exposed (BED and BED+E) or not (Ctr) to a 24-day protocol consisting of three 8-day cycles of intermittent food restriction. One week prior to the initiation of the protocol, all rats were fed standard chow ad libitum. Each cycle began with a 60% restriction of the standard food intake for four consecutive days (days 1–4), followed by a four-day period of unrestricted feeding (days 5–8). During the first two days of each refeeding period, the BED and BED+E groups received access to HCF for 2 h during the light phase, between 13:00 and 15:00 h. In the third cycle (days 21–24), no HCF was administered. On day 25 (test day), rats remained under unrestricted feeding conditions. At 13:00 h on the test day, rats from all groups were subjected to a 15-min frustration stress, during which HCF was placed in their cages but kept out of reach. During this period, rats were able to see and smell the HCF but could not access it. Following this stress exposure, all groups (Ctr, BED, and BED+E) were allowed access to HCF for 2 h, after which they returned to standard chow ad libitum until electrophysiological recordings were performed (Figure 1).

To evaluate the effect of aerobic exercise, rats in the BED+E group were housed in plastic cages (50 × 40 × 20 cm) equipped with a rodent running wheel. From the first day of BED induction until the final day of the experiment, rats had continuous access to the wheel and were allowed to run freely.

### 2.3. Electrophysiological Procedure

Rats were anesthetized with ketamine (90 mg/kg) and xylazine hydrochloride (10 mg/kg) administered intraperitoneally (i.p.). The superior cervical ganglion (SCG), including the sympathetic cervical trunk (SCT) and postganglionic nerves, was carefully exposed, dissected, and transferred to a recording chamber containing oxygenated Ringer-Krebs solution (in mM: NaCl, 136; KCl, 4; KH_2_PO_4_, 1; CaCl_2_, 2; MgCl_2_, 1; NaHCO_3_, 12; glucose, 11; pH 7.4) supplemented with 2 µM atropine. The preganglionic (SCT) and postganglionic (internal carotid nerve) fibers were stimulated and recorded using glass suction electrodes. The preganglionic nerve was stimulated with supramaximal square pulses (10–20 V, 0.2 Hz, 0.1 ms duration) delivered by a Grass S88 stimulator (Grass Instruments, Quincy, MA, USA). Compound action potentials (CAPs) from the postganglionic nerve were recorded using an extracellular differential amplifier (DP-301, Warner Instruments, Hamden, CT, USA). Signals were digitized via a data acquisition system and analyzed using custom software developed in the laboratory with LabVIEW, v21 (National Instruments, Austin, TX, USA). To induce ganglionic long-term potentiation (gLTP), nicotinic transmission was partially blocked (60–70%) with hexamethonium (100 μM), followed by the application of a supramaximal stimulus train (40 Hz, 3 s). Results were expressed as the ratio ΔR/R_0_, where ΔR = R_i_ − R_0_, with R_i_ representing the CAP amplitude at time t = i, and R_0_ the average baseline CAP amplitude recorded during the 5-min period following hexamethonium application and prior to high-frequency stimulation. LTP was evaluated by fitting the data to the function f(t) = αe^−t/τ1^ + ce^−t/τ2^. Two parameters were used to compare gLTPs: (1) LTP decay that corresponds to gLTP duration, defined as the time point at which f(t) = 0.2 (i.e., the time elapsed until the response decayed to 20% above baseline). (2) The extent of LTP that reflects gLTP magnitude quantified as the area under the potentiated response curve from t = 0 until LTP decay.

### 2.4. Immunohistochemistry

After deep anesthesia with sodium pentobarbital (125 mg/kg i.p.), rats were transcardially perfused with ice-cold phosphate-buffered saline (PBS; 0.01 M, pH 7.4), followed by cold fixative solution containing 4% paraformaldehyde in 0.1 M PBS (pH 7.4). Following perfusion, the SCG was dissected, desheathed, post-fixed in paraformaldehyde, and cryoprotected in 30% sucrose. Tissue was frozen at −20 °C and sectioned longitudinally at 12 µm thickness using a cryostat (Leica CM1520; Wetzlar, Germany). Sections were mounted onto gelatin-coated Superfrost™ slides (Electron Microscopy Sciences, Hatfield, PA, USA), and 40–45 sections per ganglion were collected along the z-axis. A random subset of these was selected for staining. Tissue sections were washed with 0.1 M PBS for 10 min, then permeabilized and blocked for 3 h at room temperature with a solution of 0.1 M PBS containing 0.3% Triton X-100 (PBS-Tx) and 10% normal donkey serum. Sections were incubated for 16 h at room temperature in a humid chamber with the following primary antibodies diluted in blocking solution (5% normal donkey serum, 5% bovine serum albumin, and 0.3% Triton X-100): goat polyclonal anti-vesicular acetylcholine transporter (VAChT; Immunostar, Hudson, WI, USA; Cat # 24286; 1:200) and mouse monoclonal anti-L-glutamic acid decarboxylase (GAD67, the enzyme responsible for GABA synthesis; Millipore-Sigma, Burlington, MA, USA; Cat # MAB5406; 1:200). After incubation, sections were washed twice in PBS-Tx (15 min each), then sequentially incubated with the following secondary antibodies: donkey anti-mouse IgG-CY3 (Jackson ImmunoResearch, West Grove, PA, USA; Cat # 715-165-151; 1:500) and donkey anti-goat IgG-Alexa 488 (Jackson ImmunoResearch; Cat # 715-585-150; 1:200), each for 2 h at room temperature. Following each incubation, sections were washed twice for 15 min in PBS-Tx and mounted with fluorescence mounting medium (Dako, Santa Clara, CA, USA).

Fluorescence was visualized using a Nikon Eclipse E600 epifluorescence microscope (Nikon Corporation, Tokyo, Japan) equipped with filter sets for CY3 and Alexa 488.

### 2.5. Image Acquisition and Analysis

Selected sections observed under the epifluorescence microscope were imaged using a Nikon A1R+ laser scanning confocal system, coupled to an Eclipse Ti-E inverted microscope (Nikon Corporation, Tokyo, Japan), equipped with a motorized stage (TI-S-E, Nikon) and controlled via NIS-Elements C software, version 5.00. Tissue sections were examined using a Plan Apo lambda 20X objective (numerical aperture 0.75). Single-plane confocal images were acquired sequentially using standard galvanometric scanners with excitation wavelengths of 488 nm and 561 nm, modulated via an acousto-optic tunable filter (AOTF). To identify specific immunoreactive puncta, we measured optical density (OD) using the Metamorph image analysis system (v. 7.5.6; Universal Imaging Corporation, Molecular Devices, Downingtown, PA, USA). Puncta were considered positive if their OD exceeded the background level (i.e., OD > background mean + 2 standard deviations). A mask (template) was generated based on these detected puncta to define specifically labeled varicosities. For each marker (VAChT and GAD67), we quantified the number of positive pixels within varicosities across the entire ganglion section. The area occupied by each marker was expressed as a percentage of the total section area. The degree of colocalization between VAChT and GAD67 was assessed by calculating the proportion of varicosities coexpressing both markers relative to the total number of GAD67-immunoreactive varicosities. Varicosities labeled only with GAD67 were considered segregated and were used to calculate the percentage of segregation.

### 2.6. Statistics

All data are presented as mean ± standard error of the mean (SEM). We used independent two-tail Student’s *t* test for HCF consumption and gLTP comparisons and one-way analysis of variance (ANOVA), followed by Tukey’s post-hoc test for comparisons of neurotransmitter expression. Differences were considered statistically significant when *p* < 0.05.

## 3. Results

### 3.1. Protocol of Cycles of Restricted Feeding and Access to HCF Induced the Experimental Model of Binge-like Eating—Exercise Prevented Installation of the Model

**Figure 2:**
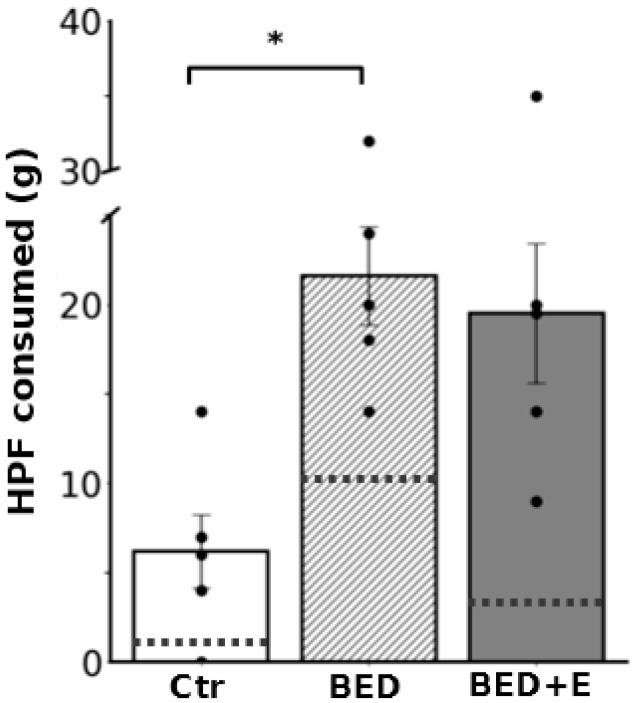
Animals that developed the model of binge eating disorder consumed more high caloric food than the control group and, in less time, than the group with exercise. Bar graphs showing the amount of HCF consumed by the three groups, control (Ctr) binge eating disorder sedentary (BED) plus exercise (BED+E). BED group consumed more HCF than Ctr (* *p* = 0.02). Doted lines indicate the percentage of HCF consumed in the first 15 min of the 2 h access, BED group consumed approximately 50% of the HCF while Ctr and BED+E consumed less than 20%; *n* = 5.

Rats subjected to repeated cycles of restricted and free feeding, followed by access to high-caloric food (HCF) and exposure to frustration stress, developed eating behavior consistent with binge-eating disorder (BED). The effectiveness of the BED induction protocol was confirmed by the significant increase in HCF consumption on the test day. Rats in the BED group consumed 21.6 ± 3.1 g of HCF during the 2-h period, in contrast to 6.2 ± 2.3 g consumed by control rats (*p* = 0.02; Figure 2).

In contrast, rats in the BED+E group (exposed to the same BED protocol but with access to voluntary exercise) did not exhibit the same binge-like pattern. Although their total HCF intake during the test (19.5 ± 5.6 g) was similar to that of the BED sedentary group (*p* = 0.9), the temporal distribution of consumption differed markedly. The BED group consumed approximately 50% of the HCF within the first 15 min, whereas the BED+E group distributed their intake more evenly over the 2-h period, indicating prevention of binge-like behavior (Figure 2).

### 3.2. Ganglia from Binge-Eating Rats Failed to Express gLTP

To evaluate ganglionic long-term potentiation (gLTP), we stimulated the preganglionic nerve of the SCG and recorded compound action potentials (CAPs) from the postganglionic internal carotid nerve, reflecting synaptic transmission between preganglionic varicosities and ganglionic neurons. The contribution of bypassing fibers was negligible, as over 95% of CAP amplitude was abolished by the nicotinic antagonist hexamethonium. In these synapses there is also muscarinic transmission that was blocked with the muscarinic antagonist atropine.

**Figure 3:**
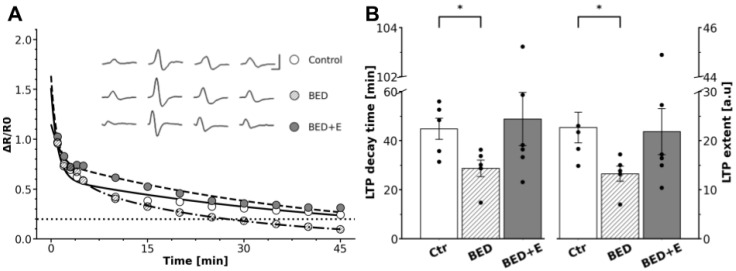
Dysautonomia associated with BED impairs LTP expression in the rat superior cervical ganglion (SCG), exercise protects against this impairment. (**A**) Representative time course of synaptic potentiation of ganglionic transmission expressed as ΔR/R_0_ in control (○), BED (Ø) and BED+E rats (●). The dotted line indicates the baseline. Inset: normalized compound action potential (CAP) recordings from the three groups of rats obtained before the train and at 5 s, 5 min, and 40 min post-train (scale bars: 10 ms, 2 mV). (**B**) Bar plots (mean ± SEM, individual values) showing that gLTP was reduced in BED rats, but not in BED+E. LTP decay time decreased from 45 ± 5 min in controls to 29 ± 3 min in BED rats (* *p* = 0.03), and LTP extent dropped from 23 ± 3 a.u. to 13 ± 2 a.u (* *p* = 0.04). LTP decay 49 ± 12 min and extent 22 ± 5 in BED+E were no different from those in the control group (*p* = 0.78), decay and (*p* = 0.9) extent. *n* = 5 per group.

In control rats, a high-frequency train of supramaximal stimuli (40 Hz, 3 s) applied to the preganglionic nerve induced a gLTP, characterized by an LTP decay of 45 ± 5 min and an LTP extent of 23 ± 4 [a.u.]. In contrast, SCG from binge-eating rats exhibited impaired gLTP expression. Postrain potentiation was significantly diminished, with a reduced LTP decay of 29 ± 3 min (*p* = 0.03) and an LTP extent of 13 ± 2 [a.u.] (*p* = 0.04; Figure 3).

### 3.3. Exercise Prevents LTP Loss

While ganglia from sedentary BED rats failed to express typical gLTP, those from rats with access to voluntary aerobic exercise (BED+E group) retained the capacity to express LTP. High-frequency stimulation of the preganglionic nerve in BED+E rats induced a gLTP which LTP decay 49 ± 12 min and extent 22 ± 5 [a.u] did not differ to the gLTP values from control animals 45 ± 5 min decay (*p* = 0.78) and 23 ± 4 [a.u.] extent (*p* = 0.9; Figure 3).

### 3.4. BED-Associated Dysautonomia Enlarged GABA Presence and Did Not Change ACh and Segregation

**Figure 4:**
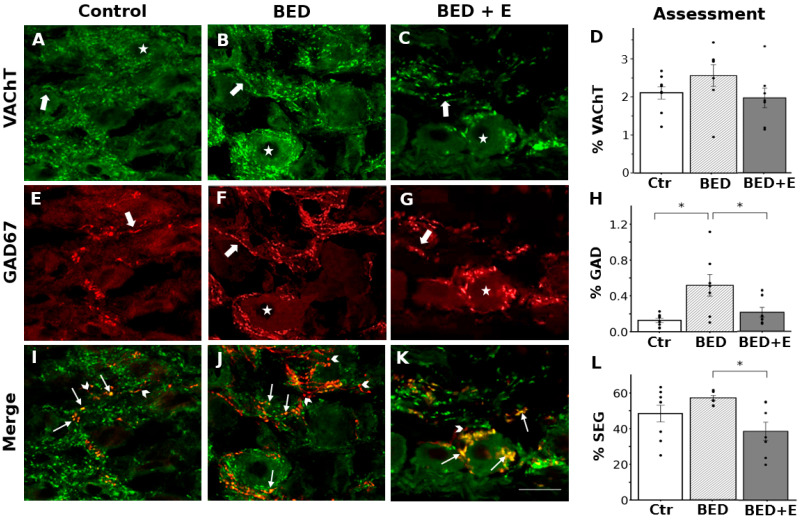
Dysautonomia associated with binge eating disorder (BED) increases GABA presence in the superior cervical ganglia (SCG), exercise protects of this increase and reduces ACh-GABA segregation from BED. Micrographs showing the same field of sections from control (**A**,**E**,**I**), BED (**B**,**F**,**J**) and BED+E rats (**C**,**G**,**K**). VAChT- and GAD67-positive varicosities were detected surrounding neuronal cell bodies (asterisks) or coursing between them (arrows). VAChT-positive varicosities were more abundant than GAD67-positive varicosities. Merged images (**I**,**J**,**K**) of VAChT (green) and GAD67 (red) immunostaining; GAD67 labeling appeared segregated (red, arrowheads) or colocalized with VAChT (yellow, small arrows). (**D**,**H**,**L**) Bar plots (mean ± SEM, individual values) showing the percentage of ganglionic area occupied by VAChT-positive varicosities (**D**), GAD67-positive varicosities (**H**), and the degree of VAChT–GAD67 segregation (**L**). BED rats showed a significant increase in GAD67 labeling, from 0.13 ± 0.02% in controls to 0.52 ± 0.12% in BED rats (F = 6.9, * *p* = 0.01). Exercise prevented the GAD67 labeling increase, in BED+E rats 0.19 ± 0.05% were different to BED rats (F = 6.9; * *p* = 0.03) and similar to control group (F = 6.9; *p* =0.83). VAChT labeling was unchanged in the three conditions (VAChT: 2.2 ± 0.2% in controls; 2.6 ± 0.3% in BED (F = 1.1; *p* = 0.5); and 2.1 ± 0.3% in BED+E (F = 1.1; *p* = 0.4). Segregation did not change between control and BDE groups, (48.5 ± 5.0% in control vs. 56.8 ± 1.4% in BED; F = 4.8; *p* = 0.4) but it reduced in BED+E group, 37.3 ± 4.9% (F = 4.8; * *p* = 0.02). *n* = 8 per group. Calibration bars: 20 μm.

Consistent with our previous studies, we found immunoreactivity for VAChT and GAD67 in bead-like structures characteristic of preganglionic varicosities, confirming the presence of cholinergic and GABAergic terminals in the SCG. Using simultaneous double immunolabeling, we identified both single-labeled and double-labeled varicosities, indicating the presence of both colocalization and segregation between VAChT and GAD67. VAChT-positive varicosities, as expected, were uniformly distributed throughout the ganglion, covering 1.2 to 3.5% of the total ganglionic area. Most were located around the somata of ganglionic neurons. In contrast, GAD67-immunoreactive varicosities were detected in a lower proportion, heterogeneously distributed, and covered only 0.04 to 0.19% of the ganglionic area. These were observed both surrounding neuronal somata and forming long interstitial fibers within the neuropil near ganglionic cells (Figure 4).

Image analysis showed no significant difference in VAChT-positive area between control and BED rats (2.2 ± 0.2% vs. 2.6 ± 0.3%, F = 1.1; *p* = 0.5; Figure 4). In contrast, the GAD67-positive area significantly increased in BED rats (0.13 ± 0.02% in control vs. 0.52 ± 0.12% in BED; F = 6.9; *p* = 0.005; Figure 4).

Analysis of VAChT and GAD67 colocalization revealed substantial segregation in both groups. A non-significant trend toward increased segregation was observed in BED rats compared to controls (48.5 ± 5.0% in control vs. 56.8 ± 1.4% in BED; F = 4.8; *p* = 0.4; Figure 4).

### 3.5. Exercise Prevents GABA Upregulation and Reduces ACh-GABA Segregation

In ganglia from rats with access to voluntary aerobic exercise during BED model induction, significant changes in GAD67 expression and VAChT-GAD67 segregation were observed compared to sedentary BED rats.

VAChT-positive varicosity area remained unchanged between BED+E and BED groups (2.6 ± 0.3% vs. 2.1 ± 0.3%, F = 1.1; *p* = 0.38). However, GAD67-positive area in BED+E rats was reduced by 63%, decreasing from 0.52 ± 0.05% in BED to 0.19 ± 0.05% in BED+E (F = 6.9; *p* = 0.03). A significant 34% reduction in VAChT-GAD67 segregation was also observed in BED+E rats compared to sedentary BED animals (37.3 ± 4.9% in BED+E vs. 56.8 ± 1.4% in BED; F = 4.8; *p* = 0.02; Figure 4).

## 4. Discussion

In this study, we reproduced the induction of a BED model in rats, which involves cycles of intermittent food restriction, ad libitum feeding, access to highly palatable caloric food, and a final period of frustration stress [24]. Rats that developed this BED phenotype exhibited signs of dysautonomia, characterized by impaired synaptic plasticity, specifically an inability to express gLTP, as well as alterations in neurotransmitter presence and in the balance between co-localization and segregation of neurotransmitters at SCG synapses, including an increased presence of GABA. In addition, our results support the beneficial role of exercise in the management of eating disorders such as BED. Aerobic exercise during model induction prevented the development of BED, the loss of gLTP, and the GABA increase. Moreover, exercise reduced ACh–GABA segregation compared with sedentary BED rats.

While gLTP alterations have been documented in several pathological models—such as spontaneously hypertensive rats [3,25], diabetic and hypertensive obese Zucker rats [26]), streptozotocin-induced diabetic rats [27]), and MS rats [28] there is a notable gap in the literature regarding direct evaluation of gLTP in rodent models of binge eating. Given this lack of data, we addressed this unexplored aspect in the present work.

Previously, we demonstrated a lack of gLTP expression in SHR [3] and in high-sucrose diet–fed rats that developed MS [28]. In SHR, as in the present study, we observed an increase in GABA [3], which led us to propose that enhanced GABAergic inhibition is responsible for gLTP failure. Supporting this hypothesis, blockade of GABA-A receptors with bicuculline restores gLTP [3]. Similarly, the increased number of GABAergic preganglionic varicosities detected in SCG from binge-eating rats in this study suggests that the lack of gLTP might be due to augmented GABAergic inhibition.

Both our group and others have reported a role for BDNF in gLTP expression [27,29], and reductions in this neurotrophin have been documented in BED rats [30,31]. Accordingly, a hypothesis that may account, at least in part, for the lack of gLTP observed in BED animals is a decrease in BDNF. Other unexplored aspects of BED-associated dysautonomia include changes in neurotransmitter expression, specifically in their presence and in the co-localization/segregation balance between transmitters. We have reported such alterations in dysautonomia associated with hypertension, stress, aging, and MS [3,6,7,28]. The present data reveal a robust increase in GABA-positive preganglionic varicosities in the SCG of binge-eating rats, which might result in sympathetic hypoactivity. Regarding the dysregulation of ANS detected in BED, as we mentioned above, findings are not conclusive, thus, there are reports of increase in sympathetic activity in human BED [13,23] and in animal models [32], other studies, consistent with our findings, have reported reduction in sympathetic activity, both in humans [33] and in animal models [34].

The contribution of GABA to BED regulation has been well documented. Using baclofen, a GABA-B receptor agonist, it has been shown that GABA participates in modulating food intake. Some findings indicate a stimulatory effect on the binge consumption of sweet–fat food [35]. Similarly, in control rats, baclofen has been found to stimulate food consumption [36]. Considering that virtually all studies on the effects of GABA, including those using baclofen, have been conducted in the central nervous system, our findings provide new evidence for GABA–BED interaction at the peripheral level. We propose that the observed increase in GABA, together with the absence of gLTP, reflects the presence of BED-associated dysautonomia, characterized by reduced sympathetic activity.

Exercise has been used to protect against eating disorders in animal models. Two different training modalities have been employed: voluntary and non-voluntary exercise. In the first, wheel running is generally used, and treadmill training in the second. We decided to assess voluntary exercise because it has shown more beneficial effects than non-voluntary exercise [37,38]. Accordingly, we found that rats that underwent the BED induction protocol but also had ad libitum access to voluntary aerobic exercise did not develop autonomic alterations. These animals consumed HCF gradually during the final exposure period, over the course of two hours, in contrast to sedentary rats, which exhibited binge eating and consumed approximately half of the food within the first 15 min. We also found that exercise prevented gLTP failure and the increase in GABA expression. Additionally, and unexpectedly, ACh-GABA segregation was reduced in exercised rats compared with sedentary binge-eating animals.

The preventive effects of voluntary physical exercise on BED onset in animal models have been reported by other groups [20,38,39,40,41]. Consistent with our findings, animals with free access to running wheels consumed less HCF during model establishment and at the final test than sedentary controls. Furthermore, exercise has been shown to reverse decreases in BDNF and its downstream plasticity-related effectors in rats fed a high-fat diet [39]. Voluntary exercise also modulates hypothalamic gene expression, reducing orexigenic and increasing anorexigenic neuropeptides [20]. Likewise, voluntary wheel running prevents HCF preference and enhances leptin signaling and function, as well as the ability of an enkephalinergic agonist to stimulate HCF preference [38,40]. In our case, the persistence of gLTP, the prevention of GABA upregulation, and the reduction in neurotransmitter segregation induced by exercise can be interpreted as a protective effect on synaptic plasticity and neurotransmitter expression.

## 5. Conclusions

A BED model was established in rats using a protocol of intermittent free-feeding/restriction cycles combined with HCF consumption and a final period of frustration stress. Rats that developed this BED phenotype exhibited dysautonomia, characterized by impaired gLTP expression and an increased presence of the inhibitory co-transmitter GABA. Rats with ad libitum access to voluntary exercise during model induction did not develop BED and, as expected, maintained gLTP expression and did not show an increase in GABA.

Our data indicate that, in addition to alterations in the commonly used indicators for detecting and characterizing dysautonomia, changes in neuroplasticity and neurotransmitter expression may also serve as useful markers for assessing disturbances in other functional properties of the autonomic nervous system. Considering the multiple roles of the ANS, characterizing additional dysfunctions may help to better explain the symptomatology of patients with BED and clarify its systemic implications. Equally important, our findings support the beneficial role of physical exercise in attenuating the autonomic and synaptic alterations associated with BED.

Our findings imply that BED is not merely a bad habit but a condition that alters neuroplasticity, including reduced synaptic potentiation, increased expression of specific neurotransmitters, and altered patterns of neurotransmitter segregation. Exercise acts as a powerful non-pharmacological intervention, preventing both the behavioral symptoms and the underlying neural changes. Thus, as practical implications for human daily living, for example, impaired neuroplasticity can result in alterations in the ability to learn or train new habits, and skewing the balance toward excessive inhibitory or excitatory signaling can lead to irritability, poor impulse control, or persistent cravings. Finally, the protective effect of exercise works like routine upkeep that helps preserve proper autonomic function.

## Data Availability

The original data presented in the study are openly available within the article. The raw data supporting the conclusions of this article will be made available by the authors on request.
